# *Potentilla rugulosa* Nakai Extract Attenuates Bisphenol A-, S- and F-Induced ROS Production and Differentiation of 3T3-L1 Preadipocytes in the Absence of Dexamethasone

**DOI:** 10.3390/antiox9020113

**Published:** 2020-01-28

**Authors:** Sun-Il Choi, Jong Seok Lee, Sarah Lee, Wan-Sup Sim, Young-Cheul Kim, Ok-Hwan Lee

**Affiliations:** 1 Department of Food Science and Biotechnology, Kangwon National University, Chuncheon 24341, Korea; docgotack89@hanmail.net (S.-I.C.); simws9197@naver.com (W.-S.S.); 2 National Institute of Biological Resources, Incheon 22689, Korea; jslee001@korea.kr (J.S.L.); lsr57@korea.kr (S.L.); 3 Department of Nutrition, University of Massachusetts Amherst, MA 01003, USA; yckim13@gmail.com

**Keywords:** endocrine disrupting chemicals, bisphenol A, Lipid metabolism disorders, ROS production, *Potentilla rugulosa* Nakai

## Abstract

Endocrine disrupting chemicals (EDCs) disrupt the physiological metabolism, thus playing an important role in the development of obesity. EDCs, the so-called ‘obesogens’, might predispose some individuals to gain weight. This study investigated the effects of bisphenol A (BPA) and its alternatives (BPS and BPF) on adipocyte differentiation and the effects of the leaves of *Potentilla rugulosa* Nakai extract (LPE) as a functional food ingredient on obesogen-induced lipid production and adipogenesis in 3T3-L1 cells. The results showed that LPE has high total phenolic and flavonoid contents (77.58 ± 0.57 mg gallic acid equivalents (GAE)/g and 57.31 ± 1.72 mg quercetin equivalents (QE)/g, respectively). In addition, LPE exerted significant antioxidant effects in terms of DPPH radical scavenging activity, reducing power, ferric-ion reducing antioxidant power, and oxygen radical absorbance capacity. BPA, BPS, and BPF increased lipid accumulation, protein expressions of adipogenic transcription factors (PPAR-γ, C/EBP-α, and aP2), and reactive oxygen species (ROS) production in 3T3-L1 cells. However, LPE suppressed the BPA-, BPS-, and BPF-induced effects on adipogenesis. Therefore, LPE has potential as a functional food supplement that can prevent bisphenol-induced lipid metabolism disorders.

## 1. Introduction

The increasing incidence of obesity is a serious global public health concern. The causes of obesity are over-ingestion, sedentary lifestyle, changing eating habits, and genetic factors. However, these factors alone do not completely support the current rise in obesity. Interestingly, Baillie-Hamilton [1] proposed a correlation between the obesity epidemic and increase in synthetic chemical production. Also, the production and use of synthetic resins increased rapidly in parallel with the increase in diabetes epidemic during the same period [2]. This correlation, coupled with experimental evidence demonstrating that certain environmental pollutants induce adipogenesis and weight gain in experimental models, led to the environmental obesogen hypothesis that posits a causative role of synthetic chemicals in the pathogenesis of obesity [3]. Thus, a new concept of “obesogen”, meaning obesity-induced environmental hormone that affects obesity, has emerged [4]. Obesogens can be defined as chemicals that functionally improperly regulate metabolic dysfunctions and promote adipogenesis and lipid accumulation [5].

Representative obesogens applied to foods are bisphenols, phthalates, and parabens [6]. Parabens are commonly used in food and beverage preservatives [7], and phthalates are used as industrial plasticizers of polyvinyl chloride that is used in plastic food packaging [8]. Bisphenols studied in this paper is a monomer of polycarbonate plastic and epoxy resin and is used as a basic raw material for the production of polycarbonate plastic and resin [9]. However, bisphenol A (BPA) has been reported to cause subfertile, metabolic disorder, obesity and diabetes [10,11,12,13] and its use is strictly restricted [14]. With the regulations related to BPA use, new alternatives to BPA have been widely explored; BPA substitutes such as bisphenol S (BPS) and bisphenol F (BPF), which have physical properties similar to those of BPA, have been developed [15]. However, due to the similarity in the chemical structures of BPA, BPS, and BPF, the role of these alternatives in endocrine disruption is still questionable.

*Potentilla rugulosa* Nakai studied herein grows in rocky crevices of mountains throughout south Korea. It has been used as an oriental medicine for promotion of hemostasis and curing of symptoms such as fever because it serves as an antioxidant [16]. However, its exact mechanism of action in metabolic diseases has never been elucidated. In this study, we investigated the antioxidant activity and BPA-, BPS-, and BPF-induced obesity effects of the leaves of *Potentilla rugulosa* Nakai extract (LPE).

## 2. Materials and Methods

### 2.1. Chemicals and Reagents

Dulbecco’s modified Eagle’s medium (DMEM), trypsin-ethylenediaminetetraacetic acid (EDTA), phosphate-buffered saline (PBS), bovine serum (BS), and fetal bovine serum (FBS) were purchased from Gibco (Gaithersburg, MD, USA). 1,1-Diphenyl-2-picrylhydrazyl radical (DPPH), quercetin, gallic acid, Folin-Ciocalteu phenol reagent, sodium carbonate, penicillin-streptomycin, and *N*-acetyl-l-cysteine (NAC) were obtained from Sigma-Aldrich Co. (Saint Louis, MO, USA).

### 2.2. Sample Preparation

LPE powder was prepared as follows: Leaves of *Potentilla rugulosa* Nakai were collected from Yeongwol-gun, Gangwon-do, Republic of Korea, in July 2016 (NIBRGR0000597649) and were identified by Dr. Jong Seok Lee at the National Institute of Biological Resources (Incheon, Korea). Coarse, dried, and grounded samples (100 g) were extracted for 24 h with 1000 mL 70% ethanol for the extraction of both fat-soluble and water-soluble compounds. The extracted material was filtered (Whatman, No. 3, Maidstone, Kent, UK) and concentrated with a rotary evaporator (EYELA, N-3000, Tokyo, Japan). Finally, the extracted material was subsequently dried using a freeze dryer (Ilshin Biobase Co., LTD, Yangju, Korea).

### 2.3. Total Phenolic and Flavonoid Contents

To determine the total phenolic content, the sample dissolved in 100% DMSO solution (1 mL) was mixed with 10% Folin-Ciocalteu reagent (1 mL) in a test tube. After 3 min, 2% sodium carbonate solution (1 mL) was added to the mixture. After the reaction mixture was kept for 40 min in the dark at 25 °C, the absorbance was measured at 750 nm (SpectraMax i3; Molecular Devices, Sunnyvale, CA, USA). A calibration curve was constructed with different concentrations of gallic acid [(mg GAE)/g].

To determine the total flavonoid content, the sample dissolved in 100% DMSO solution (0.5 mL) was incubated with 95% ethanol (1.5 mL), 10% aluminum chloride (0.1 mL), 1 M potassium acetate (0.1 mL), and distilled water (2.8 mL) at 25 °C for 60 min. The absorbance was measured at 420 nm, and a calibration curve was constructed with different concentrations of quercetin [(mg QE)/g].

### 2.4. Antioxidant Activity

DPPH radical scavenging activity of LPE was determined as follows: sample solutions (0.2 mL) were mixed with 0.4 mM DPPH solution (0.8 mL; yielding an absorbance of 1.0 ± 0.1 at 515 nm) and incubated at 25 °C for 60 min. The absorbance measured at 490 nm and the radical scavenging activity was calculated using Equation (1):

Radical scavenging activity (%) = (1 − A_experiment_/A_control_) × 100 (1)

In reducing power assay, sample solutions (0.5 mL) were mixed with 1% potassium ferricyanide (2.5 mL) and 0.2 M sodium phosphate buffer (2.5 mL). After incubation at 50 °C for 20 min, 10% trichloroacetic acid (2.5 mL) was added to the reaction mixtures. The mixtures were centrifuged at 1790× *g* for 10 min and the supernatant (2.5 mL) was mixed with 0.1% iron chloride (0.5 mL) and distilled water (2.5 mL). The absorbance was measured at 700 nm.

In ferric-ion reducing antioxidant power (FRAP), the 300 mM sodium acetate buffer, 10 mM 2,4,6-Tripyridyl-s-triazine (TPTZ), and 20 mM ferric chloride were mixed in a ratio of 10:1:1 to prepare the FRAP reagent. Next, 50, 100 and 200 μg/mL of sample solutions (0.05 mL) were incubated with the FRAP reagent (1.5 mL) and distilled water (0.15 mL) at 37 °C for 4 min. The absorbance measured at 593 nm.

In oxygen radical absorbance capacity (ORAC) assay, the phosphate buffer (75 mM; pH 7.4) was used for preparation of sample solutions and reagents. The sample solution (25 μL) was mixed with 40 nM fluorescein (150 μL) and 18 mM AAPH (25 μL). The plates were incubated for 15 min at 37 °C, and fluorescence of the sample solutions was measured using a fluorescence microplate reader (Spectramax Gemini EM; Molecular Devices, Sunnyvale, CA, USA) at emission and excitation wavelengths of 520 and 485 nm. The results were obtained through the trolox calibration curve and the area under the fluorescence decay curve. ORAC values were expressed as the area under the curve (AUC), which was determined using Equation (2):

AUC = 1 + f1/f0 + f2/f0 + f3/f0 + f4/f0 + … f/31/f0(2)

where f(0) is the initial fluorescence values and f(n) is the fluorescence value measured every 3 min.

### 2.5. Cell Culture and Differentiation

3T3-L1 preadipocytes were purchased from the American Type Culture Collection (#CL-173; Manassas, VA, USA). The cells were plated at 37 °C in 5% CO_2_ atmosphere and grown in DMEM with 10% BS, 3.7 g/L sodium bicarbonate, and 1% P/S until confluence. To induce differentiation, 2-days post-confluence preadipocytes (day 0) were cultured with a hormonal cocktail as adipogenic inducers (0.5 mM IBMX, 1.0 µM DEX, and 1.0 µg/mL insulin; MDI) in 10% FBS medium. After 2 days, the culture medium was changed to a fresh medium containing only 1.0 µg/mL insulin in 10% FBS medium and replenished every 2 days until differentiation till 10 days. BPA, BPS, and BPF were treated 6 days before preadipocyte differentiation was maintained during cell differentiation until the cells were harvested.

### 2.6. Cell Proliferation

The cell proliferation was measured using the 2,3-bis(2-methoxy-4-nitro-5-sulfophenyl)-2H-tet-razolium-5-carboxanilide (XTT) assay (WelGene, Seoul, Korea). 3T3-L1 preadipocytes were seeded on each well of 48-well plates (1 × 10^4^ cells/well). BPA, BPS, and BPF were treated for 6 days before preadipocyte differentiation. The cells were differentiated with an induction medium with BPA, BPS, BPF, and LPE for 10 days. After incubation, N-methyl dibenzopyrazine methyl sulfate (PMS) and XTT reagent were added to each well, followed by incubation at 37 °C for 4 h in 5% CO_2_ atmosphere. The absorbance was measured at 450 nm and the cell viability was calculated using Equation (3):

Viability = (A study group/A of control group) × 100%(3)

### 2.7. Oil Red O Staining Assay

Adipocyte differentiation was progressed for 10 days to measure the lipid production by BPA, BPS, and BPF and the inhibitory effect of LPE on 3T3-L1 cells. To fix the adipocyte, 10% formaldehyde was treated at 24 °C for 1 h and washed with 500 μL of 60% isopropanol solution to completely dry the cells. The completely dried cells were stained with 0.6% Oil Red O solution at 24 °C for 1 h. The Oil red O combined lipid component was eluted with 100% isopropanol and the absorbance was measured at 490 nm.

### 2.8. Nitroblue Tetrazolium (NBT) Assay and Flow Cytometry

In NBT assay, adipocyte differentiation was progressed for 10 days to evaluate the ROS production by BPA, BPS, and BPF and the inhibitory effect of LPE in 3T3-L1 cells. Adipocytes were incubated with 0.2% NBT solution at 24 °C for 90 min and formazan was eluted with 50% acetic acid. The absorbance measured at 593 nm.

Adipocytes were incubated with 10 μM H_2_DCFDA at 24 °C for 30 min. FACS Calibur flow cytometer (BD Biosciences, San Jose, CA, USA) was used to measure the fluorescence (excitation and emission wavelength is 488 and 545 nm, respectively). Data analysis was based on 10,000 detected events using Cell Quest software 6.0 (BD Biosciences).

### 2.9. Western Blot Analysis

The protein lysis buffer (0.1% SDS, 1% NP-40, 150 mM NaCl, 0.25% sodium deoxycholate, 1 mM pepstatin A, and 1 mM PMSF) was used for the lysis of the cells, and the lysates were centrifuged at 4 °C for 20 min at 12,000× *g*. The protein concentrations were quantified using the Bradford protein assay kit (Bio-Rad Laboratories, Inc., Hercules, CA, USA) and 30 μg of protein was subjected to SDS-PAGE. The loaded gel was transferred onto a polyvinylidene difluoride membrane, and TBST with 5% skimmed milk were used to block. The membrane was incubated with primary antibodies at 4 °C overnight and secondary antibodies at 24 °C for 1 h. The primary antibodies specific for β-actin (1:1000; cat. 4967), adipocyte protein 2 (aP2; 1:1000; cat. 3544), CCAAT/enhancer-binding protein-α (C/EBP-α; 1:1000; cat. 2295), peroxisome proliferator-activated receptor-γ (PPAR-γ; 1:1000; cat. 2443) and the secondary antibodies (1:3,000; cat. 7076) were purchased from Cell Signaling Technology (Danvers, MA, USA). The proteins were detected using the ECL detection reagent (PowerOpti-ECL; BioNote, Inc., Seoul, Korea) and imaged by Chemi Doc image software 5.2.1.

### 2.10. Statistical Analysis

In this study, all experiments were performed over three times. Using Duncan’s multiple range tests and ANOVA, all data were statistically analyzed. The value of *p* < 0.05 was considered statistically significant (SAS Institute, Inc., Cary, NC, USA).

## 3. Results

### 3.1. Phenolic and Flavonoid Contents

The total polyphenol and flavonoid contents of LPE were measured, as shown in Table 1. Phenolic and flavonoid compounds are found in almost all parts of plants including leaves, flowers, fruits, stems, and roots [17]. They have antioxidant effects and protect the DNA, cell proteins, and enzymes in the body [18]. The results showed that LPE contained 77.58 ± 0.57 mg GAE/g of total polyphenol and 57.31 ± 1.72 mg QE/g of total phenolic compounds. These results are in good agreement with those of another study by Tomczyk et al. [19], who determined total phenolic content of seven other species of the *Potentilla* genus (*P. fruticose*, *P. grandiflora*, *P. norvegica*, *P. thuringiaca*, *P. pensylvanica*, *P. crantzii*, and *P. nepalensis*). The content of the total polyphenols ranged from 49.9 ± 1.5 mg GAE/g for the aerial parts of *P. pensylvanica* to 116.3 ± 3.9 mg GAE/g for *P. fruticosa*. Therefore, these results indicated that AFE, compared with the extracts of other the *Potentilla* species, contained high levels of phenolic and flavonoids compounds.

### 3.2. Effect of LPE on Antioxidant Activity

**Figure 1:**
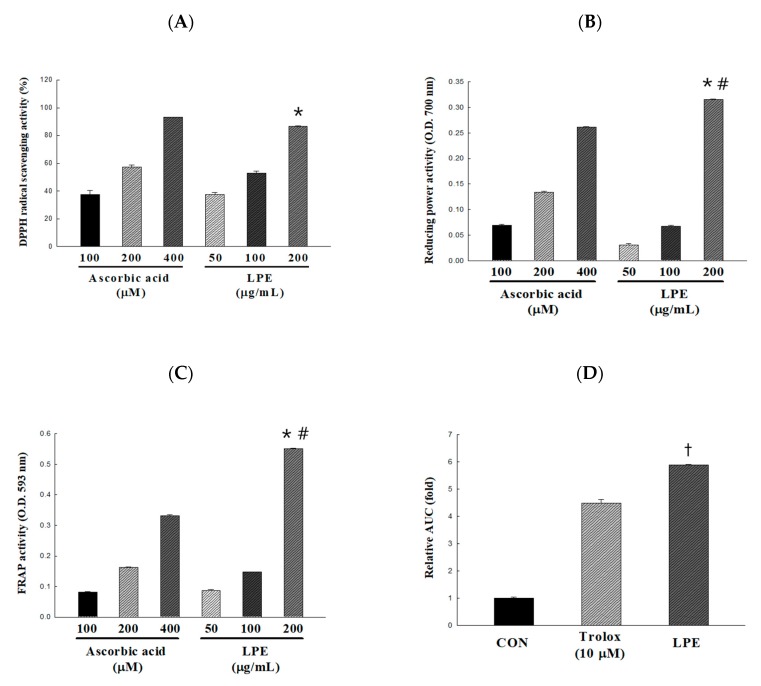
Antioxidant activity of the *Potentilla rugulosa* Nakai ethanolic extract (LPE) was established based on (**A**) DPPH radical-scavenging activity, (**B**) reducing power, (**C**) Ferric-ion reducing antioxidant power (FRAP) activity, and (**D**) areas under the curve fluorescence decay curve (AUC) value for ORAC. All values are presented as the mean ± SD. * *p* < 0.05 VS. 100 mg/mL of LPE; ^#^ *p* < 0.05 VS. 400 μM of ascorbic acid; ^†^ *p* < 0.05 VS. 10 μM of trolox, according to one-way analysis of variance.

In this study, the antioxidant activity of LPE extracts was evaluated by various in vitro antioxidant activity assays. The concentration of LPE was set to the maximum concentration at which the reaction was not saturated up to 200 μg/mL. The DPPH radical scavenging activity of LPE was dose-dependent, a scavenging activity of 37.37 ± 1.59, 52.97 ± 1.42, and 86.60 ± 0.27% was obtained with 50, 100, and 200 μg/mL of LPE, respectively (Figure 1A). In addition, 100 μg/mL LPE showed a similar radical scavenging activity as 200 μg/mL ascorbic acid. The reducing power of 50, 100, and 200 μg/mL of LPE, the absorbance values (700 nm) increased significantly to 0.03, 0.07, and 0.32, respectively (Figure 1B). The FRAP of LPE was dose-dependent, and absorbance values (593 nm) of 0.09, 0.15, and 0.55 were obtained with 50, 100, and 200 μg/mL of LPE, respectively (Figure 1C). In particular, 200 μg/mL of LPE had a significantly higher reducing power than 400 mM ascorbic acid. The areas under the curve fluorescence decay curve (AUC) profiles were calculated to assess the effectiveness of the samples [20]. As shown in Figure 1D, the AUC value increased by 5.89 ± 0.03-fold following LPE (2 μg/mL) treatment compared to the no-treatment control. ORAC values were expressed as the area AUC, which was determined using Equation (2). The ORAC value of LPE was 6941.92 ± 43.20 μmol trolox equivalent (TE)/g.

### 3.3. Effect of BPA, BPS, and BPF on Cell Viability and Lipid Accumulation

**Figure 2:**
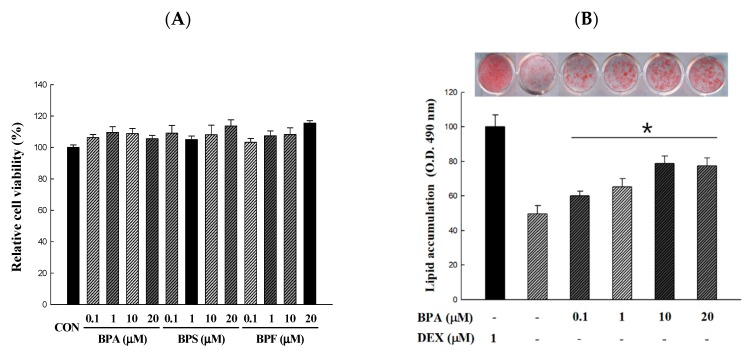

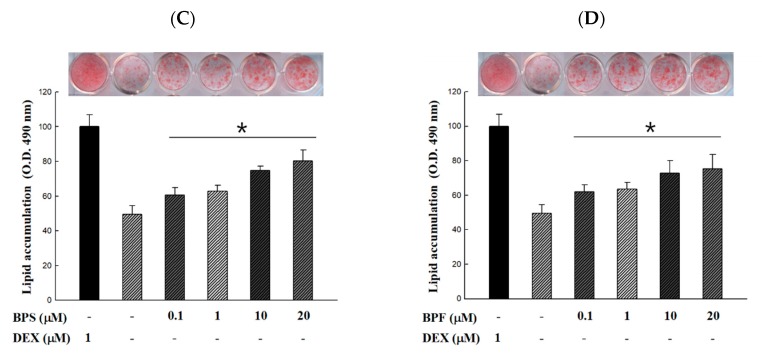
Cell viability and lipid accumulation from Bisphenol A (BPA), Bisphenol S (BPS), and Bisphenol F (BPF)-induced 3T3-L1 adipocyte. 3T3-L1 adipocytes were differentiated for 10 days with different concentrations of bisphenols. BPA, BPS, and BPF were treated 6 days before preadipocytes differentiation and maintained during cell differentiation. (**A**) Effect of BPA, BPS, and BPF on the viability was determined by an XTT assay and the absorbance at 450 nm was measured. (**B**–**D**) The accumulated lipids were stained with Oil Red O reagent and the absorbance at 490 nm was measured. All values are presented as the mean ± SD. * *p* < 0.05 VS. MI treatment group, according to one-way analysis of variance.

The XTT assay was used to determine the cytotoxicity and concentration range of BPA, BPS, and BPF suitable for treatment during the period from 3T3-L1 preadipocytes to adipocytes. The cytotoxicity of various concentrations of BPA, BPS, and BPF was expressed as a percentage compared to the MDI control group. As shown in Figure 2A, BPA, BPS, and BPF had no any cytotoxic effect at 0.1, 1, 10, and 20 μM. In addition, cell morphology changes were not observed under the microscope (data not shown). We confirmed that lipid production was induced by BPA, BPS, and BPF at concentrations of 0.1 to 20 μM, which showed no toxicity (Figure 2B–D). The control group was divided into the MDI (0.5 mM IBMX, 1.0 µM DEX, and 1.0 µg/mL insulin) treated group and the MI treated group (except dexamethasone). In addition, BPA, BPS, and BPF were treated for 6 days before differentiation and then differentiated with MI for 10 days. Lipid accumulation was expressed as a percentage compared to the 1.0 µM dexamethasone-treated control group. Treatments with BPA, BPS, and BPF during adipogenic differentiation resulted in a significant increase in the amount of lipid accumulated compared with that in the MI control group. Moreover, 20 µM of the BPA, BPS, and BPF treated groups were not significantly different from the MDI treatment group (77.53 ± 4.38, 80.25 ± 6.22, and 75.39 ± 8.37%, respectively).

### 3.4. Effect of LPE on Cell Viability and Lipid Accumulation in BPA, BPS, and BPF-Induced 3T3-L1 Adipocyte

**Figure 3:**
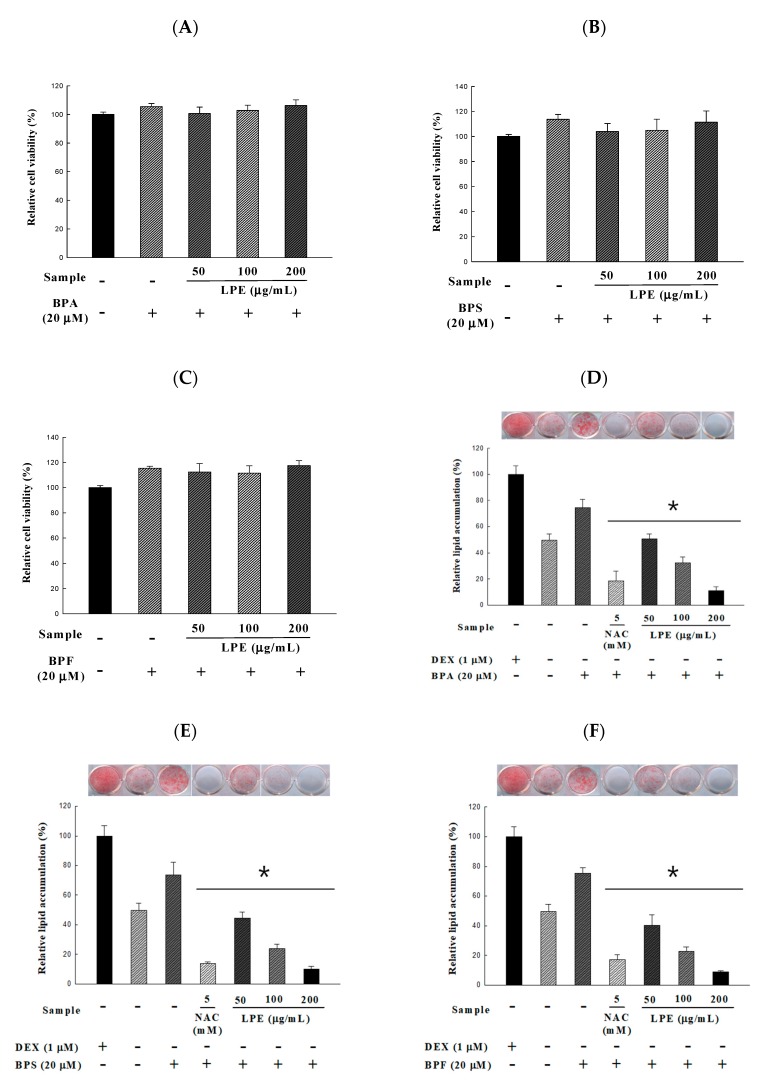
Effects of *Potentilla rugulosa* Nakai ethanolic extract (LPE) on BPA, BPS, and BPF-induced 3T3-L1 adipocyte in (**A**–**C**) cell viability and (**D**–**F**) lipid accumulation were determined by an XTT assay and ORO staining. 20 μM of BPA, BPS and BPF were treated 6 days before preadipocytes differentiation and maintained during cell differentiation. Various concentrations of LPE were dissolved in DMSO and treated on 3T3-L1 adipocyte (added on day 0 of differentiation) for 10 days. All values are presented as the mean ± SD. * *p* < 0.05 VS. MI treatment group, according to one-way analysis of variance.

In this study, we investigated whether LPE could regulate the BPA-, BPS-, and BPF-induced differentiation of 3T3-L1 preadipocytes into mature adipocytes. Prior to measuring the lipids accumulated, we confirmed the cytotoxicity of various concentrations of LPE (50, 100, and 200 μg/mL). As indicated in Figure 3A–C, all concentrations of LPE treated with 20 μM of BPA, BPS, and BPF showed no cytotoxicity compared to the no-treatment control group. Thus, these concentrations of BPA, BPS, BPF, and LPE were chosen and used in all other experiments. To determine the effect of LPE on lipid accumulation in BPA-, BPS-, and BPF-induced 3T3-L1 adipocyte differentiation (Figure 3D–F), all groups were treated with MI (0.5 mM IBMX and 1.0 µg/mL insulin). Also, we used NAC as a positive control, which is widely known to reduce ROS production and lipid accumulation. The treatment of 3T3-L1 cells with 20 μM of BPA, BPS, and BPF caused lipid accumulation, whereas all concentrations of LPE significantly decreased lipid accumulation to a level comparable to the BPA, BPS, and BPF treatment group. In addition, 200 μg/mL of LPE treated groups showed no significant differences from the NAC treatment groups in inhibiting lipid accumulation.

### 3.5. Effect of LPE on ROS Production in BPA, BPS, and BPF-Induced 3T3-L1 Adipocyte

**Figure 4:**
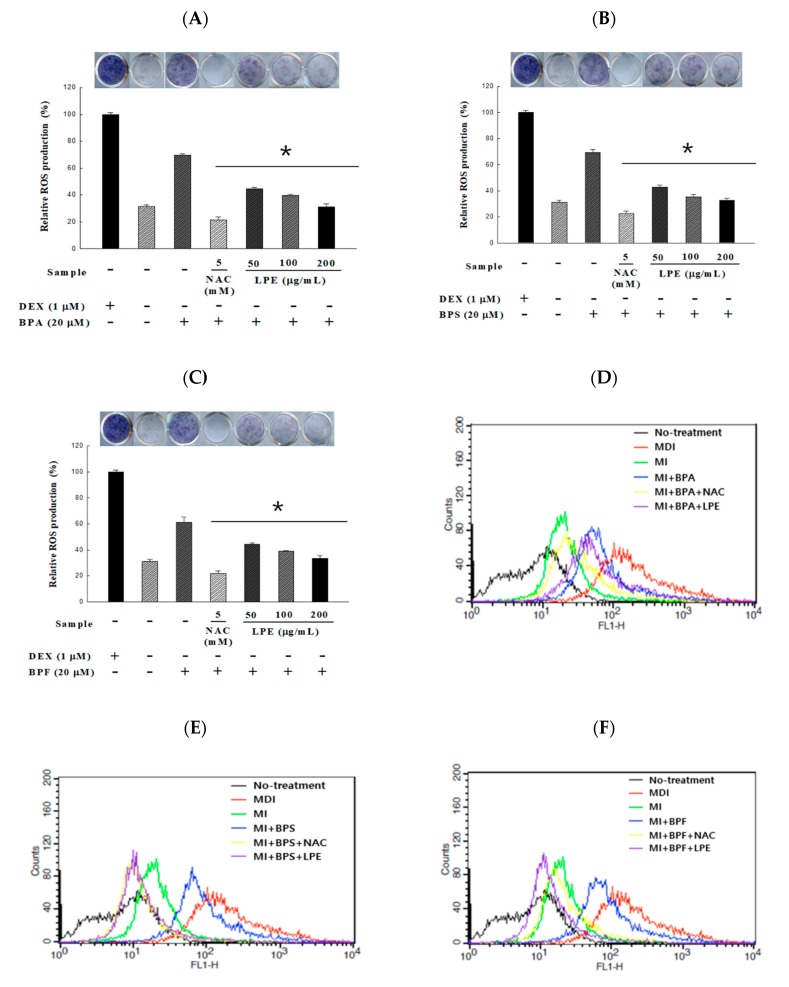
Effects of LPE on BPA-, BPS-, and BPF-induced 3T3-L1 adipocyte in reactive oxygen species (ROS) were determined. After 10 days of 20 μM of bisphenol-induced adipocyte differentiation, ROS was measured by (**A**–**C**) colorimetric analysis using NBT assay. Adipocyte were incubated with an NBT solution for 90 min, and the absorbance was measured at 593 nm. Another method of measuring ROS is (**D**–**F**) fluorescence analysis using DCFDA assay. Adipocytes were incubated with 10 μM H_2_DCFDA for 30 min and the fluorescence was measured using flow cytometry. All values are presented as the mean ± SD. * *p* < 0.05 VS. MI treatment group, according to one-way analysis of variance.

The reduction effect of LPE on ROS production during BPA-induced adipocyte differentiation was determined by the NBT and DCFDA assays. In order to confirm the inhibitory effects of ROS production of LPE, the groups were divided in the same way as in the lipid accumulation experiment. In the absence of dexamethasone, BPA, BPS, and BPF treatment significantly increased ROS-induced dark formazan production. All concentrations of LPE treated groups significantly decreased the BPA, BPS, and BPF induced production of dark formazan. Interestingly, all of the 200 µg/mL LPE treatment groups showed no significant differences from the MI treatment group (Figure 4A–C). Further, we confirmed ROS production using H_2_DCFDA. The fluorescence of DCF increased to 98% during adipocyte differentiation with MDI compared to the untreated H_2_DCFDA group. In addition, the fluorescence of DCF increased to 9% in the MI-treated group, and 76, 89, and 87% in the MI groups co-treated with 20 µM of BPA, BPS, and BPF, respectively. However, the increased fluorescence was significantly reduced in the 200 µg/mL LPE treatment groups.

### 3.6. Effect of LPE on the Expression of BPA, BPS and BPF-Induced Adipogenic Transcription Factors

**Figure 5:**
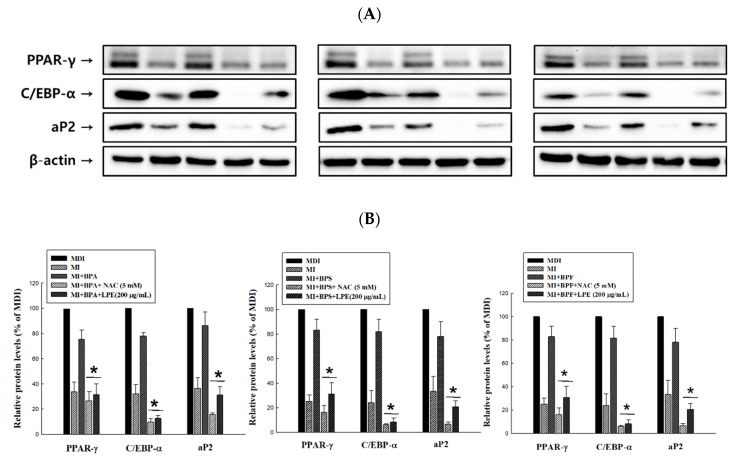
Effects of LPE on the protein expressions of BPA-, BPS-, and BPF-induced adipogenic transcription factors (PPAR-γ: 53, 57kDa, C/EBP-α: 42 kDa and aP2: 15kDa) in 3T3-L1 adipocytes. (**A**) Total protein was obtained using a lysis buffer and quantified by loading, transfer, blocking, and appropriate antibody reactions. (**B**) Expression levels of adipogenic transcription factors were densitometrically normalized to those of β-actin and compared to the MDI control group as a percentage in each antibody. All values are presented as the mean ± SD. * *p* < 0.05 VS. BPA, BPS, or BPF treatment group, according to one-way analysis of variance.

To determine whether LPE reduces the BPA-, BPS-, and BPF-induced adipogenic transcription factors (PPAR-γ, C/EBP-α and aP2) in 3T3-L1 adipocytes, a western blot analysis was conducted. As shown in Figure 5A,B, all the adipogenic transcription factors significantly increased with BPA, BPS, and BPF treatment. Even all the adipogenic transcription factors treated with BPS, aP2 with BPA, and C/EBP-α with BPF showed no significant differences from the MDI treated group. However, the increased protein expression levels were significantly reduced in the 200 µg/mL LPE treatment groups.

## 4. Discussion

Endocrine disrupting chemicals exposed to the external environment interfere with normal endocrine system functions and unlike natural hormones, they are not easily degraded and are concentrated in fat tissues of the human body [21]. Several hypotheses have confirmed that damage can even be transmitted to descendants [22]. Therefore, they were identified as factors that have a profound effect on human health as well as ecosystem stability and have received increasing interest from academics, the government, and the general public [23]. BPA is a monomer used in the production of plastics and epoxy resins, as well as in the manufacture of food packaging materials including cans, and milk bottles; thus, individuals are easily exposed to it in daily life [24]. Evidentially, many studies have reported that BPA can be detected in human biological samples such as breast milk, urine, serum, pregnancy-associated fluids, semen, and follicular fluid [25]. BPA has estrogen activity and is known to be toxic to reproductive and immune systems and can lead to the formation of DNA adducts [26,27,28]. In addition, BPA is stored in adipose tissues and can cause significant effects such as adipocyte differentiation, lipogenesis, adipokine release [29,30,31,32]. Therefore, the use of BPA has been regulated and especially banned in infant products [33]. New materials to replace BPA are being developed, and representative BPA substitutes include BPS and BPF, which have physical properties similar to BPA [15]. In fact, these substitutes are used to manufacture ‘BPA-free’ products [34]. However, these alternatives have also been reported to lead to endocrine disruption [35,36]. Therefore, this study attempted to discover food ingredients that suppress endocrine disruption owing to obesogen.

The genus of *Potentilla* plants are a member of the Rosaceae family, which is mainly distributed in the temperate, alpine zones of the Northern hemisphere [37]. The biological activities of these plants have been studied and certain common species have been considered important in traditional medicine. Evidence from pharmacological studies has suggested that extracts from the aerial and/or underground parts of genus *Potentilla* have antioxidant, hypoglycemic, anti-inflammatory, and antitumor properties [38,39,40,41]. Most of the biological effects of the *Potentilla* species can be explained by the high amount of hydrolysable and condensed tannins, flavonoids, and triterpenes present in all parts of the plant [42]. In this study, we determined the total phenolic and flavonoid contents of LPE as 77.58 ± 0.57 mg GAE/g and 57.31 ± 1.72 mg QE/g, respectively. These results are in good agreement with those of another study by Tomczyk et al. [19], who determined total phenolic content of seven other species of the *Potentilla* genus (*P. fruticose*, *P. grandiflora*, *P. norvegica*, *P. thuringiaca*, *P. pensylvanica*, *P. crantzii*, and *P. nepalensis*). The content of the total polyphenols ranged from 49.9 ± 1.5 mg GAE/g for the aerial parts of *P. pensylvanica* to 116.3 ± 3.9 mg GAE/g for *P. fruticosa*. Therefore, these results indicated that AFE, compared with the extracts of other the *Potentilla* species, contained high levels of phenolic and flavonoids compounds. In addition, we confirmed the high antioxidant capacity of LPE by the DPPH radical scavenging activity, reducing power, FRAP, and ORAC assay (Figure 1). Phenolic compounds have phenolic hydroxyl groups, are known to exhibit high antioxidant activity, and are highly correlated with each other [43,44]. Owing to these effects, *P. rugulosa* is considered to have a wide applicability as a functional food supplement.

In general, obesity is known to be caused by the accumulation of fat tissues owing to hypertrophy and hyperplasia of newly produced adipocytes through adipogenesis [45]. Adipogenesis of 3T3-L1 preadipocytes is involved in growth arrest, mitotic clonal expansion (MCE), and terminal differentiation, and coordinated by transcription factor [46]. Moreover, the activity of key adipogenic transcription factors is regulated by endocrine hormones such as the growth and thyroid hormone, glucocorticoids, estrogen, and androgen [47]. Obesogens promote adiposity by changing programming of fat cell development (adipogenesis), increasing energy storage in fat tissues, and interfering with neuroendocrine control of appetite and satiety [48]. Many studies have reported that BPA mimics the action of estrogen and glucocorticoid hormones [49]. In this study, lipid accumulation and adipogenic transcription factors were shown to increase by BPA, BPS, and BPF treatment with no treatment with dexamethasone. However, these actions of obesogen were significantly inhibited by the treatment of LPE. Adipocyte differentiation is characterized by the production of ROS with increased mitochondrial metabolism. Increased ROS levels provide a permissive oxidative environment for signaling that initiates cellular differentiation [50]. Our data showed that LPE inhibited BPA-, BPS-, and BPF-induced ROS production by NBT and DCFDA assay. It was therefore suggested that LPE is beneficial to obesogen-induced metabolic disorder in murine 3T3-L1 cells. However, the present study did not distinguish the mechanism of obesogen-induced adipogenesis and the LPE inhibition effect involved with antioxidants. In a future study, we will analyze specific components to demonstrate the effectiveness of LPE.

## 5. Conclusions

To summarize, this study demonstrated that BPA, BPS, and BPF induced adipogenesis of murine preadipocytes occurs through the activation of adipogenic transcription factors (PPAR-γ, C/EBP-α, and aP2) and increased lipid and ROS production. However, LPE, which clearly exhibits antioxidant properties, suppresses these effects of BPA, BPS, and BPF in 3T3-L1 cells. Thus, the potential of LPE as a functional food ingredient was demonstrated and new insights into the molecular basis underlying the adipogenic properties of environmental pollutants such as EDCs were obtained.

## Figures and Tables

**Table 1:** Quantification of antioxidant components in LPE.

Compound Class	Amount
Total phenolic contents	77.58 ± 0.57 mg GAE ^1)^/g
Total flavonoid contents	57.31 ± 1.71 mg QE ^2)^/g

^1)^ GAE: gallic acid equivalent, ^2)^ QE: quercetin equivalent.

## Abbreviations

EDCs	Endocrine disrupting chemicals
BPA	Bisphenol A
BPS	Bisphenol S
BPF	Bisphenol F
LPE	Leaves of *Potentilla rugulosa* Nakai extract
ROS	Reactive oxygen species
FRAP	Ferric-ion reducing antioxidant power
ORAC	Oxygen radical absorbance capacity
NBT	Nitroblue tetrazolium
aP2	Adipocyte protein 2
C/EBP-α	CCAAT/enhancer-binding protein-α
PPAR-γ	Peroxisome proliferator-activated receptor-γ
DEX	Dexamethasone
IBMX	3-Isobutyl-1-methylxanthine
NAC	n-acetylcysteine

## References

[1] Baillie-Hamilton P.F. (2002). Chemical toxins: A hypothesis to explain the global obesity epidemic. J. Altern. Complement. Med..

[2] Neel B.A., Sargis R.M. (2011). The paradox of progress: environmental disruption of metabolism and the diabetes epidemic. Diabetes.

[3] Grün F., Blumberg B. (2009). Endocrine disrupters as obesogens. Mol. Cell Endocrinol..

[4] Grun F., Blumberg B. (2007). Perturbed nuclear receptor signaling by environmental obesogens as emerging factors in the obesity crisis. Rev. Endocr. Metab. Disord..

[5] Legeay S., Faure S. (2017). Is bisphenol A an environmental obesogen?. Fund Clin. Pharmacol..

[6] Veiga-Lopez A., Pu Y., Gingrich J., Padmanabhan V. (2018). Obesogenic endocrine disrupting chemicals: identifying knowledge gaps. Trends Endocrinol. Metab..

[7] Wong K.H., Durrani T.S. (2017). Exposures to endocrine disrupting chemicals in consumer products - a guide for pediatricians. Curr. Probl. Pediatr. Adolesc. Health Care.

[8] Ibarra V.G., de Quirós A.R.B., Losada P.P., Sendón R. (2018). Identification of intentionally and non-intentionally added substances in plastic packaging materials and their migration into food products. Anal. Bioanal. Chem..

[9] Cooper J.E., Kendig E.L., Belcher S.M. (2011). Assessment of bisphenol A released from reusable plastic, aluminium and stainless steel water bottles. Chemosphere.

[10] Hond E.D., Tournaye H., De Sutter P., Ombelet W., Baeyens W., Covaci A., Cox B., Nawrot T.S., Larebeke N.V., D’Hooghe T. (2015). Human exposure to endocrine disrupting chemicals and fertility: A case–control study in male subfertility patients. Environ. Int..

[11] Magueresse-Battistoni B.L., Labaronne E., Vidal H., Naville D. (2017). Endocrine disrupting chemicals in mixture and obesity, diabetes and related metabolic disorders. World J. Biol. Chem..

[12] Carwile J.L., Michels K.B. (2011). Urinary bisphenol A and obesity: NHANES 2003–2006. Environ. Res..

[13] Alonso-Magdalena P., Quesada I., Nadal A. (2011). Endocrine disruptors in the etiology of type 2 diabetes mellitus. Nat. Rev. Endocrinol..

[14] Song C.Y., Kim W., Gye M.C. (2017). Current state of use and the risks of bisphenols: A minireview. Korean J. Environ. Biol..

[15] Le Fol V., Aït-Aïssa S., Sonavane M., Porcher J.M., Balaguer P., Cravedi J.P., Zalko D., Brion F. (2017). In vitro and in vivo estrogenic activity of BPA, BPF and BPS in zebrafish-specific assays. Ecotox. Environ. Safe.

[16] Choi J., Lee S.E., Lee J.H., Kim G.S., Noh H.J., Kim S.Y. (2014). *Persicaria orientalis* and *Potentilla fragarioides* extracts inhibit NF-κB translocation and nitric oxide production in LPS-stimulated RAW 264.7 Cells. J. Appl. Biol. Chem..

[17] Hertog M.G., Hollman P.C., Venema D.P. (1992). Optimization of a quantitative HPLC determination of potentially anticarcinogenic flavonoids in vegetables and fruits. J. Agric. Food Chem..

[18] Solimani R., Bayon F., Domini I., Pifferi P.G., Todesco P.E., Marconi G., Samori B. (1995). Flavonoid-DNA interaction studied with flow linear dichroism technique. J. Agric. Food Chem..

[19] Tomczyk M., Pleszczyńska M., Wiater A. (2010). Variation in total polyphenolics contents of aerial parts of Potentilla species and their anticariogenic activity. Molecules.

[20] Bisby R.H., Brooke R., Navaratnam S. (2008). Effect of antioxidant oxidation potential in the oxygen radical absorption capacity (ORAC) assay. Food Chem..

[21] Jackson E., Shoemaker R., Larian N., Cassis L. (2011). Adipose tissue as a site of toxin accumulation. Compr. Physiol..

[22] Daston G.P., Cook J.C., Kavlock R.J. (2003). Uncertainties for endocrine disrupters: Our view on progress. Toxicol. Sci..

[23] Lyons G. (2006). Viewpoint: Policy requirements for protecting wildlife from endocrine disruptors. Environ. Health. Perspect..

[24] Howe S.R., Borodinsky L., Lyon R.S. (1998). Potential exposure to bisphenol A from food-contact use of epoxy coated cans. J. Coat. Technol..

[25] Vandenberg L.N., Hauser R., Marcus M., Olea N., Welshons W.V. (2007). Human exposure to bisphenol A (BPA). Reprod. Toxicol..

[26] Wang P., Luo C., Li Q., Chen S., Hu Y. (2014). Mitochondrion-mediated apoptosis is involved in reproductive damage caused by BPA in male rats. Environ Toxicol. Pharmacol..

[27] Rees Clayton E.M., Todd M., Dowd J.B., Aiello A.E. (2010). The impact of bisphenol A and triclosan on immune parameters in the US population, NHANES 2003–2006. Environ. Health Perspect..

[28] Atkinson A., Roy D. (1995). In vivo DNA adduct formation by bisphenol A. Environ. Mol. Mutagen..

[29] Chamorro-Garcia R., Kirchner S., Li X., Janesick A., Casey S.C., Chow C., Blumberg B. (2012). Bisphenol A diglycidyl ether induces adipogenic differentiation of multipotent stromal stem cells through a peroxisome proliferator-activated receptor gamma-independent mechanism. Environ. Health Perspect..

[30] Lee D.H., Porta M., Jacobs D.R., Vandenberg L.N. (2014). Chlorinated persistent organic pollutants, obesity, and type 2 diabetes. Endocr. Rev..

[31] Yang M., Chen M., Wang J., Xu M., Sun J., Ding L., Lv X., Ma Q., Bi Y., Liu R. (2016). Bisphenol A promotes adiposity and inflammation in a nonmonotonic dose-response way in 5-week-old male and female C57BL/6J mice fed a low-calorie diet. Endocrinology.

[32] Hugo E.R., Brandebourg T.D., Woo J.G., Loftus J., Alexander J.W., BenJonathan N. (2008). Bisphenol A at environmentally relevant doses inhibits adiponectin release from human adipose tissue explants and adipocytes. Environ. Health Perspect..

[33] Braun J.M., Kalkbrenner A.E., Calafat A.M., Yolton K., Ye X., Dietrich K.N., Lanphear B.P. (2011). Impact of early-life bisphenol A exposure on behavior and executive function in children. Pediatrics.

[34] Grumetto L., Montesano D., Seccia S., Albrizio S., Barbato F. (2008). Determination of bisphenol A and bisphenol B residues in canned peeled tomatoes by reversed-phase liquid chromatography. J. Agric. Food Chem..

[35] Moreman J., Lee O., Trznadel M., David A., Kudoh T., Tyler C.R. (2017). Acute toxicity, teratogenic, and estrogenic effects of bisphenol A and its alternative replacements bisphenol S, bisphenol F, and bisphenol AF in zebrafish embryo-larvae. Environ. Sci. Technol..

[36] Rochester J.R., Bolden A.L. (2015). Bisphenol S and F: a systematic review and comparison of the hormonal activity of bisphenol A substitutes. Environ. Health Perspect..

[37] Eriksson T., Donoghue M.J., Hibbs M.S. (1998). Phylogenetic analysis of Potentilla using DNA sequences of nuclear ribosomal internal transcribed spacers (ITS), and implications for the classification of *Rosoideae* (Rosaceae). Plant Syst. Evol..

[38] Tomczyk M., Paduch R., Wiater A., Pleszczyńska M., Kandefer-szersze M., Szczodrak J. (2013). The influence of aqueous extracts of selected *potentilla* species on normal human colon cells. Acta. Pol. Pharm..

[39] Leporatti M.L., Ivancheva S. (2003). Preliminary comparative analysis of medicinal plants used in the traditional medicine of Bulgaria and Italy. J. Ethnopharmacol..

[40] Miliauskas G., Beek T.A., Venskutonis P.R., Linssen J.P., Waard P., Sudhölter E.J. (2004). Antioxidant activity of *Potentilla fruticosa*. J. Sci. Food Agric..

[41] Syiem D., Syngai G., Khup P., Khongwir B., Kharbuli B., Kayang H. (2002). Hypoglycemic effects of *Potentilla fulgens* L. in normal and alloxan-induced diabetic mice. J. Ethnopharmacol..

[42] Tomczyk M. (2011). Secondary metabolites from *Potentilla recta* L. and Drymocallis rupestris (L.) Soják (syn. *Potentilla rupestris* L.)(*Rosaceae*). Biochem. Syst. Ecol..

[43] Lien E.J., Ren S., Bui H.H., Wang R. (1999). Quantitative structure-activity relationship analysis of phenolic antioxidants. Free Radic. Biol. Med..

[44] McDonald S., Prenzler P.D., Antolovich M., Robards K. (2001). Phenolic content and antioxidant activity of olive extracts. Food Chem..

[45] Alessi M.C., Lijnen H.R., Bastelica D., Juhan-Vague I. (2003). Adipose tissue and atherothrombosis. Pathophysiol. Haemost. Thromb..

[46] Chang E., Kim C.Y. (2019). Natural Products and Obesity: A focus on the regulation of mitotic clonal expansion during adipogenesis. Molecules.

[47] Steinmetz R., Brown N.G., Allen D.L., Bigsby R.M., Ben-Jonathan N. (1997). The environmental estrogen bisphenol A stimulates prolactin release in vitro and in vivo. Endocrinology.

[48] Janesick A.S., Blumberg B. (2016). Obesogens: an emerging threat to public health. Am. J. Obstet. Gynecol..

[49] Sargis R.M., Johnson D.N., Choudhury R.A., Brady M.J. (2010). Environmental endocrine disruptors promote adipogenesis in the 3t3-l1 cell line through glucocorticoid receptor activation. Obesity.

[50] Tormos K.V., Anso E., Hamanaka R.B., Eisenbart J., Joseph J., Kalyanaraman B., Chandel N.S. (2011). Mitochondrial complex III ROS regulate adipocyte differentiation. Cell Metab..

